# Surgical management of raised intra-ocular tension in the hostile ocular surface - recurrent tube erosion in a patient with systemic sclerosis: a case report

**DOI:** 10.1186/s12886-018-0856-5

**Published:** 2018-09-14

**Authors:** Gurjeet Jutley, Elizabeth Yang, Phillip Bloom

**Affiliations:** Western Eye Hospital, London, UK

**Keywords:** Pars plana tube, Baerveldt tube, Tube erosion, Scleroderma, Systemic sclerosis, Glaucoma drainage surgery, Glaucoma, Autologous tissue harvesting

## Abstract

**Background:**

The surgical management of patients with uncontrolled glaucoma and scleroderma is challenging, as the hostile ocular surface poses a challenge to surgery. A serious complication is tube erosion, with the risk of subsequent endophthalmitis. Here, we present a novel technique of harvesting autologous tissue to successfully manage recurrent tube extrusion.

**Case presentation:**

MG is a 60-year-old Arabic lady diagnosed with scleroderma, that was previously managed with systemic corticosteroids. She has chronic open angle glaucoma, with a failed left eye trabeculectomy, which was then managed by a Baerveldt tube (BVT) insertion. Eight months after this primary surgery, she developed an anterior uveitis. This was further complicated by conjunctival erosion, tube exposure, leak around the sclerostomy site and hypotony. The erosion was likely secondary to her tight eyelids as a result of her scleroderma. She was taken back to theatre for tube revision, with single layer amniotic membrane transplant (AMT) over the exposed area, but the tube was eroding again after 2 months. She eventually underwent tube extraction, pars plana tube plate stabilisation, pars plana vitrectomy (PPV), pars plana tube insertion, phacoemulsification and intra-ocular lens insertion, jointly with the vitreo-retinal surgeons and with high dose prednisolone cover both pre- and post-operatively. We harvested the *capsule* which had grown over the end plate of the original tube. We sutured this over the new tube, specifically over a single layer of tutoplast prior to conjunctival closure. Almost a year on, the pars plana tube remains in place with no complications.

**Conclusions:**

This case highlights the role of a pars plana tube in cases of cicatricial disease, with the use autologous tissue instead of grafts wherever possible. In patients with systemic disease such as scleroderma, pre-operative immunosuppression helps to reduce the of erosion in difficult cases.

## Background

The surgical management of patients with uncontrolled glaucoma who have a pre-existing systemic inflammatory condition is difficult, as a hostile ocular surface poses a significant challenge to surgery. Numerous complications can arise post-operatively, the most sinister of which is tube erosion and subsequent endophthalmitis.

We present an interesting patient with a multi-systemic inflammatory condition, and describe a novel technique harvesting autologous tissue to successfully manage recurrent tube extrusion. This has not been previously described in the literature to date. We also suggest an independent indication for primary pars plana tube insertion that should be considered by all glaucoma surgeons.

## Case presentation

MG is a 60-year-old Arabic lady diagnosed with scleroderma, also known as systemic sclerosis. She is under annual observation without systemic medication by a Rheumatologist, having previously required high dose oral corticosteroids. Her past ocular history is positive for right amblyopia, which can now be attributed to a macular scar of unknown aetiology. In her adult life, she had been diagnosed with chronic open angle glaucoma, undergoing a trabeculectomy & three needlings in the right eye.

The left eye also had a failed trabeculectomy, which was then managed by a Baerveldt tube (BVT) insertion. Eight months after this primary surgery, she developed anterior uveitis with hypopyon, with both intra-vitreal and anterior chamber samples revealing no growth. Unfortunately, this was further complicated by conjunctival erosion, tube exposure, leak around the sclerostomy site and hypotony. She was taken back to theatre for tube revision, including using a tube extender and a single layer amniotic membrane transplant (AMT) over the exposed area.

### Surgical management

She was referred to the Western Eye Hospital (WEH) to answer a specific question: what should we do about the tube exposed once again through the conjunctiva in the left eye despite the revision with AMT? After much deliberation and lengthy discussions with the patient, we explored the techniques which would minimise further erosions and delivered the following options:

- Completely abort the premise of conjunctival drainage by removing the BVT and either doing:
  - ○ Endocyclophotocoagulation (ECP), hence prohibiting aqueous production.
  - ○ Assess the integrity of the angle, by completing a minimally invasive glaucoma surgery such as an iStent, hence encouraging drainage through the irido-corneal angle.
  - ○ This was unacceptable to MG, since she wanted to avoid staged procedures such as sequential ECP’s and potentially eventual tube revision.
- Move the existing tube into the ciliary sulcus, +/− iridotomy to observe its position
  - ○ Although this would not address the issue of friability of the conjunctiva overlying the tube
- Retract the tube, with a right angle clip and pass this existing tube through the pars plana
  - ○ This would circumvent completely discarding the tube, although the plate would have to be retracted back away from the precarious limbal area. The clip would extend the tube enough for it to reach the vitreous cavity.
  - ○ The disadvantage of this technique is this clip has a high profile and would potentially put undue stress on an already compromised conjunctiva by tenting it up.
- Move the plate to the supero-nasal quadrant and enter anterior chamber (AC)
- Take out original tube and put in a pars plana tube (PPT)

**Fig. 1:**
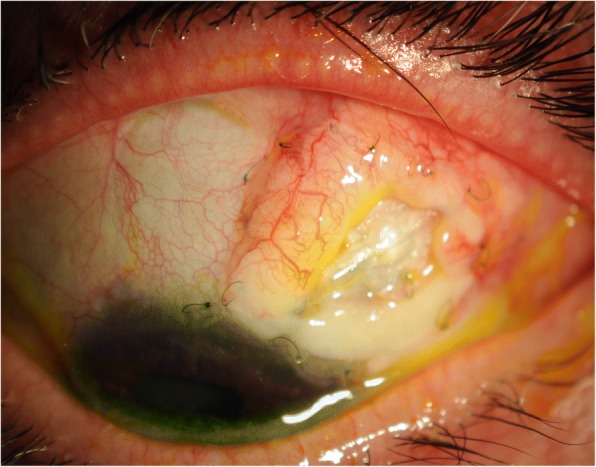
Conjunctival erosion with exposed tube extender, following first revision at WEH: double layered tutoplast and autologous conjunctival graft harvested from the infero-nasal quadrant of the ipsilateral eye

**Fig. 2:**
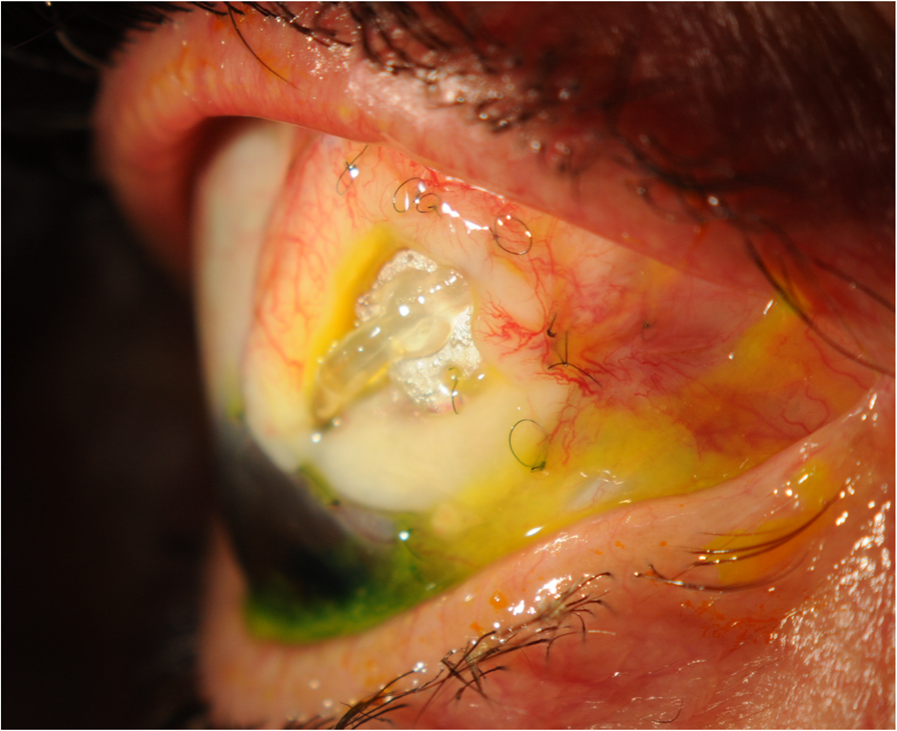
Conjunctival erosion with exposed tube extender following first revision at WEH

**Fig. 3:**
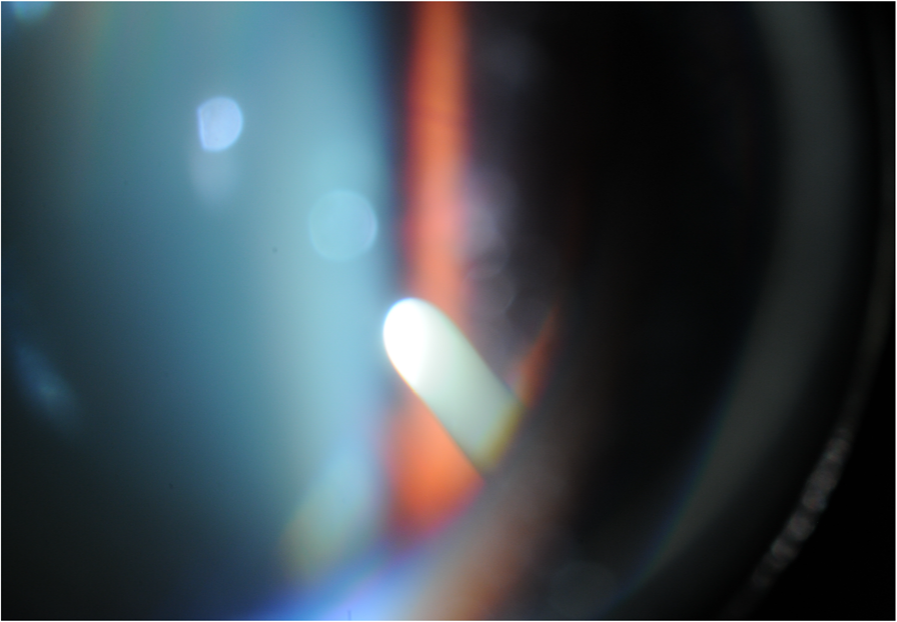
Slit-lamp photograph of the pars plana tube

We proceeded with revision involving gluing double layered pericardial tissue (tutoplast) and autologous conjunctival graft harvested from the infero-nasal quadrant of the ipsilateral eye, sutured over the graft. Despite careful follow-up, the tube was observed to be eroding through the conjunctiva once again at 2 months (Figs. 1 and 2). Further surgical intervention became inevitable and she underwent a tube extraction, pars plana tube plate stabilisation, pars plana vitrectomy (PPV), pars plana tube insertion (Fig. 3), phacoemulsification and intra-ocular lens insertion, jointly with the vitreo-retinal surgeons and with high dose prednisolone cover both pre- and post-operatively. This satisfied her request of having a definitive procedure including refractive correction and had an advantage over the right angle clip of possessing a flatter profile, as well as the plate being suitably distal from the limbus.

**Fig. 4:**
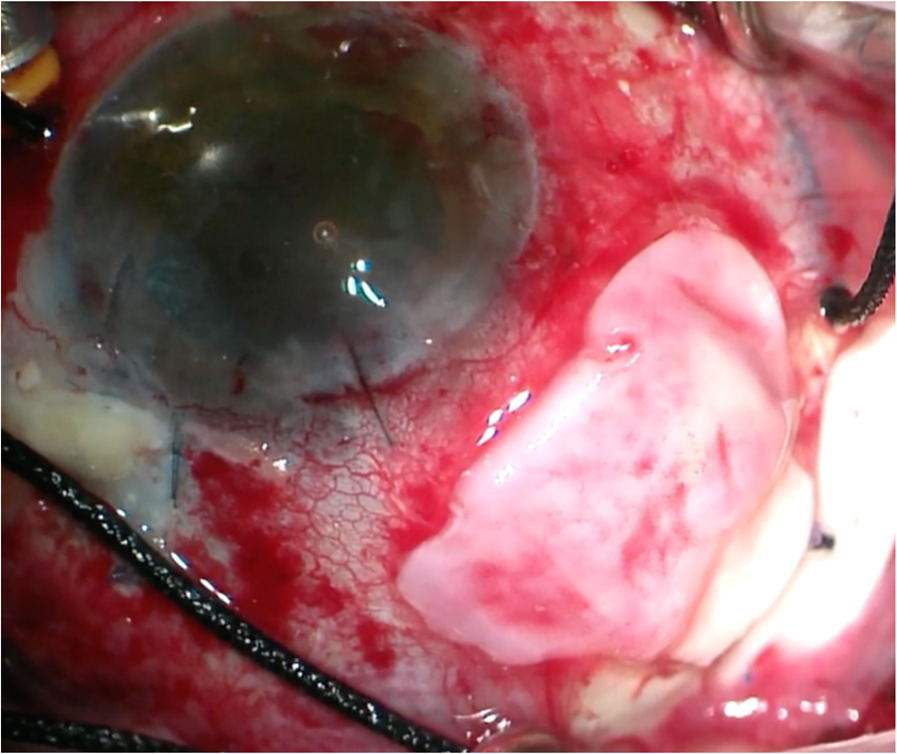
Intra-operative photograph showing the autologous capsule being harvested over the single layer of tutoplast, prior to conjunctival closure

Pre-operatively, we had multi-disciplinary team meeting to discuss the best way to circumvent future conjunctival erosion. Whilst not in the literature, we decided to harvest the *capsule* which had grown over the end plate of the original tube. We sutured this over the new tube, specifically over a single layer of tutoplast prior to conjunctival closure (see Fig. 4). The closure itself took 2 h to complete and we considered the role of fibrin glue in achieving this, as we wanted to circumvent the adherence and subsequent fibrosis it would lead to, in case a further revision would be necessary. We did proceed using glue with the premise that it assisted the dispersal of pressure, minimizing the tension on the conjunctiva with sutures alone.

## Discussion

Systemic sclerosis (SSc) is a multisystem connective tissue disease. It is characterised by fibrosis of the skin and internal organs in addition to vascular abnormalities including secondary Raynaud’s phenomenon, renal crisis and pulmonary hypertension. Internal organ involvement causes substantial morbidity and mortality. Fibroblast dysfunction, innate/adaptive immune system defects and fibroproliferative microvasculopathy have all been implicated in the pathophysiology of SSc [1]. Within the UK, SSc has an annual incidence of 3.7 per million and a prevalence of 31–88 per million, with a peak age of onset of 40–50 years [2]. Classification criteria have been developed, and major disease subgroups are recognised, particularly limited, diffuse cutaneous and overlap subsets [3]. Although the evidence appears unconvincing, patients with early diffuse cutaneous SSc (dcSSc) can be treated with a number of immunosuppressive agents including methotrexate, mycophenolate mofetil or intravenous cyclophosphamide [4, 5]. In those patients at risk of severe organ involvement autologous haematopoietic stem cell transplant may be of benefit [6].

Conjunctival erosion and tube exposure is fraught with danger for the ophthalmologist due to the risk of intra-cameral migration of microbes, leading to endophthlamitis [7]. The incidence of conjunctival and tutoplast melting has been reported up to 2–7% of all tube surgeries [8, 9]. In these cases, the use of very anterior tube extenders and covering it with tutoplast and conjunctival autograft is also likely to cause recurrent conjunctival erosion. In the first instance, in such patients with such compromised conjunctiva with poor healing, bulky tube extenders or pars plana clips should be avoided, to reduce the risk of erosion.

A variety of different options exist to aid repair of the defect, all of which encompass an exogenous substance directly covering the tube under the undermined conjunctiva. These options include autologous fascia lata, allogenic sclera, dura or tutoplast. The latter is our preferred at the Western Eye Hospital due to reduced risks of viral transmission, non-dependence on the eye bank and the availability of uniform size and quality of tissue [10]. Various authors have described in the literature other techniques to augment these adjuncts in both systemically well patients and those with immunological conditions, like SSc. Lama et al. described their experience of two *systemically well* patients presenting with tube erosion, one of whom unfortunately developed endophthalmitis [7]. One patient was managed only with a conjunctival autograft and the other had a further tutoplast, both of whom at final follow-up had no evidence of further erosion. Unfortunately, MG did not have such an outcome after this combined approach was adopted in her first surgical revision at the Western Eye Hospital. She has an inflammatory condition that has heterogenous ocular manifestations, although since SSc is so rare the evidence citing ophthalmic involvement is restricted to case reports and case series. Some reports suggest that patients with scleroderma have shallow fornices, tight eyelids, conjunctival congestion and telangiectasia, with histological biopsies revealing extensive subepithelial fibrosis [11]. We encountered difficulties in the second revision due to limited surgical space secondary to small apertures, which we managed with a lateral canthotomy intra-operatively. Our own experience correlates with the literature, in suggesting patients with variants of SSc have exquisitely friable conjunctiva. Husain et al. have recently described a patient with CREST syndrome (calcinosis, Raynaud’s phenomenon, oesophageal varices, sclerodactyl and telangiectasia) undergoing aqueous shunt surgery [12]. Pre-operatively, they sensibly identified the patient would be at high risk of scarring and bleb failure if a trabeculectomy was attempted. Intra-operatively, it was documented that conjunctival closure was particularly challenging and the post-operative period was complicated by tube erosion, necessitating revision surgery [12]. The authors used an AMT to directly cover the bare scleral area, using a combination of fibrin glue and 8.0 vicryl [12]. At six-months follow-up, there was no further tube erosion, the supramid was still in situ and the pressure was 15 mmHg off drops. AMT has also been used previously to manage these defects, with Ainsworth et al. describing a case series of systemically well patients with conjunctival erosions following Ahmed tube implantations [13]. The technique they described was an inner graft over the tutoplast, epithelial side up, with outer graft shaped larger than the conjunctival defect, with the epithelial side down [13]. The outer layer sloughed off within 2 weeks, with complete re-epithelisation within 4 months [13]. In fact, one of the patients described in the series was extremely similar to MG: having previously failed trabeculectomy, with exposure of the subsequent tube. The initial attempt of direct conjunctival closure failed, as did a secondary autologous conjunctival graft [13]. Eventually further erosions were circumvented by a scleral patch and double layered AMT, unlike MG who suffered AMT’s. Another potential adjunct to prevent excess fibrosis during healing in these eyes would be treatment with oral doxycycline, which has been shown in the literature to be effective [14],

PPT’s are perhaps underused clinically and represent an excellent choice in situations of AC abnormalities, including extensive peripheral anterior synechiae, or glaucoma with concurrent posterior segment pathology requiring PPV. Early studies were performed using Molteno, Baerveldt and Schoket tubes, with 51 out of 60 total eyes with varying aetiologies having good glaucoma control, with IOP less than 21 mmHg [15–17]. PPT have been shown to be non-inferior to anterior chamber tubes, in post vitrectomised eyes [18]. Chihara et al. showed tube surgery led to endothelial cell loss independent to physical touch, attributed to cytokines liberated from the iris [19]. Conversely, endothelial cell loss at 1 year in PPT was comparable to phacoemulsification alone, mean percentage loss at 1 year being 5.3 [19]. De Guzman et al. showed that mean IOP went from 33 pre-operatively to 13 post pars plana Molteno or Baerveldt tubes, although there was no statistical significance in reducing corneal decompensation and graft failure by doing a pars plana tube [20]. Some caution must be exhibited with reports of hypotony, retinal detachments and choroidal effusions [21]. For MG, a PPT was chosen not for the typical indications alluded to above, but rather to utilize the plate position being so distal to the limbus, where the majority of the friability of the conjunctiva exists. We believe that both this and peri-operative immunosupression are the reasons that this approach was met with success. This has been a challenging and complex case and one which the PPT approach appears to have overcome the issue of a hostile ocular surface. However, this is only one example of a successful outcome in a unique situation, and more research and experience would be required by the way of PPT in managing a complex ocular surface and recurrent erosions.

## Conclusion

**Fig. 5:**
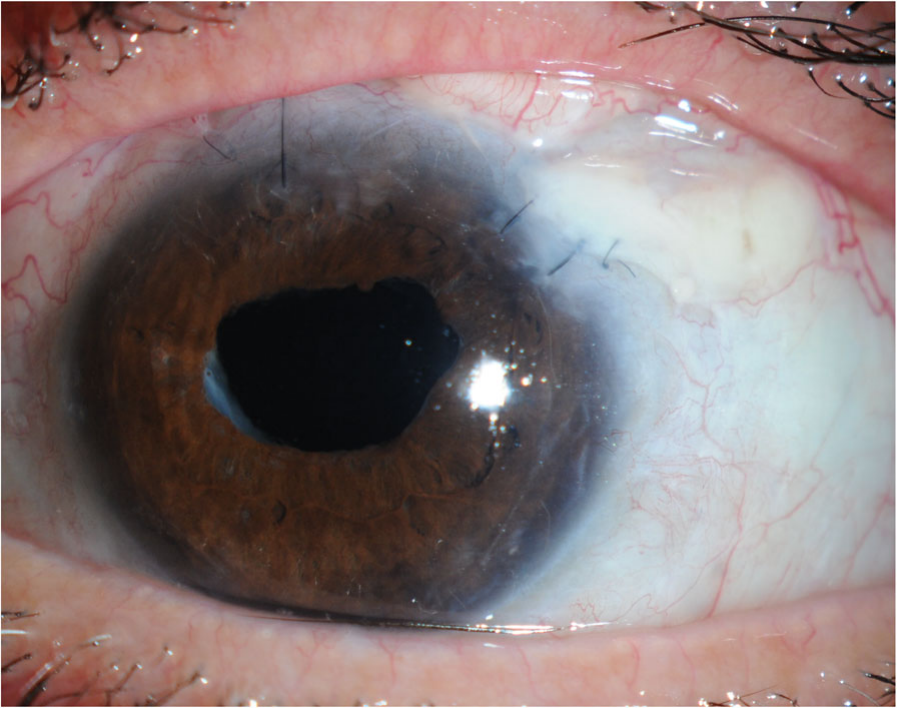
Outcome at last follow up with no evidence of conjunctival erosion

**Fig. 6:**
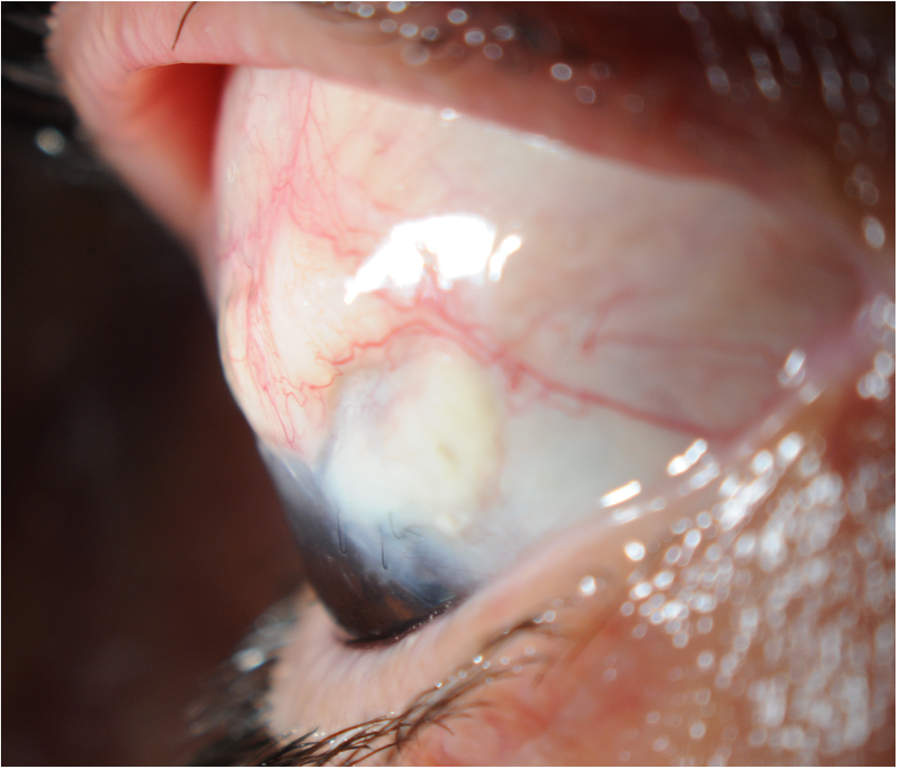
No evidence of erosion at last follow-up

This case is novel because we used an innovative technique to manage problem of recurrent erosion by initially moving it elsewhere and secondarily, after this failed, harvesting autologous tissue to augment heterologous tissue, thus successfully breaking the vicious cycle. The autologous capsular membrane enabled us to avoid harvesting donor tissue or use amniotic membrane as described previously. It has to be said that this was only possible due to her having a previous tube inserted. To her great delight, at the last follow-up after removal of the supramid suture, the IOP in the left eye was 10 and the best spectacle corrected visual acuity 6/18 (Figs. 5 and 6). Our next challenge is managing her right eye, whereby in the setting of recurrent needlings for a failing bleb, we are approaching the decision of a tube with great trepidation! In the setting of a visually significant cataract, we have opted to list her for a right phacoemulsification and ECP, liaising closely with our oculoplastic colleagues to facilitate enough space for the probe to fit in such tight lids.

Finally, we believe that a ‘hostile ocular environment’ could be considered as an indication for a primary PPT, as exemplified by this case. The tube plate is posterior from the limbus, where the majority of the friability of the conjunctiva exists. The patients that are most likely to benefit are those with:

- High risk of aqueous misdirection
- Compromised corneas
- Limited space in the AC
- Co-existing retinal pathology
- Hostile conjunctival environment

However, these are complex patients with multifactorial ocular issues, and careful surgical planning is always required. We believe this opens up an avenue for research into the use of the pars plana tube approach for these hostile ocular surfaces.

### Learning points

1. Consider PPT in cases of cicatricial conjunctivitis
2. Optimise pre-operative immunosuppression to ensure reduced risk of erosion in difficult cases
3. Use autologous tissue instead of grafts wherever possible

## Abbreviations

AC: Anterior chamber
AMT: Amniotic membrane transplant
BVT: Baerveldt tube
dcSSc: Diffuse cutaneous SSc
ECP: Endocyclophotocoagulation
IOP: Intraocular pressure
PPT: Pars plana tube
PPV: Pars plana vitrectomy
SSc: Systemic sclerosis
WEH: Western Eye Hospital
